# Jostaberry (*Ribes × nidigrolaria*) as a Functional Ingredient for 3D Printed Snacks: Impact of Starch Type and Storage on Antioxidant Profile and Stability

**DOI:** 10.3390/antiox15080971

**Published:** 2026-08-05

**Authors:** Ana Mandura Jarić, Anica Bebek Markovinović, Luna Maslov Bandić, Josipa Ljubičić, Ana Valentić, Manuela Zadravec, Ana Vulić, Boris Duralija, Sandra Zavadlav, Danijela Bursać Kovačević

**Affiliations:** 1 Faculty of Food Technology and Biotechnology, University of Zagreb, Pierottijeva 6, 10000 Zagreb, Croatia; 2 Faculty of Agriculture, University of Zagreb, Svetošimunska 25, 10000 Zagreb, Croatia; 3 Croatian Veterinary Institute, Savska Cesta 143, 10000 Zagreb, Croatia; 4 Department of Food Technology, Karlovac University of Applied Sciences, Trg J. J. Strossmayera 9, 47000 Karlovac, Croatia

**Keywords:** jostaberry, 3D food printing, phenolic stability, antioxidant capacity, starch, storage

## Abstract

The growing demand for functional foods and personalized nutrition has increased interest in three-dimensional printing (3DP) as a technology for developing customized food products. Jostaberry (*Ribes × nidigrolaria*), a fruit rich in antioxidant compounds, has rarely been explored in 3DP food applications. This study evaluated the suitability of jostaberry for producing 3DP snacks and investigated the effects of starch type (corn or wheat) and storage (12 days/4 °C) on physicochemical properties, bioactive compounds, antioxidant capacity, and microbiological stability. Both starch type and storage significantly influenced product characteristics, whereas storage was the main factor affecting stability. During storage, moisture loss, changes in total soluble solids, color differences, and reductions in organic acid concentrations were observed. Although condensed tannins and anthocyanins decreased by 27.6% and 37.3%, respectively, total phenolic content remained relatively stable, suggesting compound-specific changes rather than an overall loss of phenolics. Compared with wheat starch, corn starch resulted in higher total flavonoid and hydroxycinnamic acid contents, whereas wheat starch retained higher malic acid concentrations. Antioxidant capacity also gradually decreased during storage. Despite these changes, the 3DP snacks remained microbiologically stable and complied with food safety requirements throughout the storage period. These findings demonstrate that jostaberry is a promising functional ingredient for 3DP snacks, providing high bioactive potential and acceptable refrigerated storage stability.

## 1. Introduction

The growing consumer demand for functional foods and healthier dietary choices has driven the development of hybrid plant species designed to combine the most desirable characteristics of their parent varieties. In this context, the jostaberry (*Ribes × nidigrolaria*) was created by crossing blackcurrant (*Ribes nigrum*), valued for its high nutritional content, with gooseberry (*Ribes uva-crispa*), appreciated for its large, juicy berries [1]. The resulting hybrid is a shrub-like plant that produces spherical, dark-purple fruits with a tart and refreshing flavor. Its fruits are consumed fresh, used as flavoring agents in jams and juices, and are processed into both alcoholic and non-alcoholic beverages [2]. Due to their considerable content of bioactive compounds, antioxidants, dietary fiber, carbohydrates, vitamins, and minerals, jostaberries have high nutritional and biological value, making them a promising raw material for the development of functional foods [3].

Compared to other fruit species such as redcurrant, blackcurrant, raspberry, blackberry, and gooseberry, jostaberry has the highest total polyphenol content (1593.92 mg GAE 100 g^−1^ FW) [4]. It has a diverse phytochemical profile, with substantial levels of phenolic compounds, including catechins (95 mg 100 g^−1^ FW) and anthocyanins (31.6 mg 100 g^−1^ FW), as well as monoterpenes (31%), organic acids (28%), and vitamins (16%) [5]. These bioactive constituents contribute to the pronounced antioxidant capacity of jostaberries, indicating their potential role in mitigating oxidative stress–related disorders [3]. Moreover, the bioaccessibility of phenolics in jostaberry ranges from 69.91% to 89.07%, further highlighting its nutritional and functional potential [6].

Jostaberry fruits are not only consumed fresh but have also been investigated in several food-processing applications. Previous studies have reported their successful incorporation into jams, juices, syrups, and fruit-based beverages, where they contributed desirable sensory characteristics and enhanced antioxidant capacity. In addition, jostaberry powders and pomace have been explored as functional ingredients in bakery formulations, resulting in improved polyphenol content, higher dietary fiber levels, and reduced predicted glycemic response [2]. Different drying techniques (freeze-drying, oven-drying) have also been applied to jostaberry to obtain stable powders and extracts rich in bioactive compounds with antioxidant and antimicrobial properties [3]. Furthermore, fermented jostaberry products, including wines and mixed fruit beverages, have demonstrated the suitability of this fruit for value-added processing [1]. These findings confirm that jostaberry is a versatile and underutilized berry crop with strong potential for the development of novel functional foods and advanced processing technologies.

Despite its high nutritional and bioactive value, jostaberry remains an underutilized fruit with limited commercial presence compared to other berry species. Its seasonal availability, relatively short postharvest shelf life, and low incorporation into processed foods restrict wider consumer acceptance and market potential. Current literature provides limited information on the stability of these bioactive compounds during processing and storage, particularly in innovative food systems where mechanical and thermal stresses may accelerate degradation. In addition, little is known about the interactions between jostaberry constituents and carrier matrices, which may influence compound stability and bioaccessibility. Therefore, innovative processing approaches are needed to increase the utilization of jostaberry and convert it into attractive value-added products while preserving its functional properties. Three-dimensional (3D) food printing has emerged as a promising technology for this purpose, as it enables the transformation of fruit-based materials into customized structures with controlled shape, texture, and portion size. In addition, 3D printing can improve product attractiveness, facilitate personalization, and create new opportunities for the development of functional snacks enriched with bioactive compounds [7]. Within this context, jostaberry is a suitable raw material for novel 3D-printed foods [8]. For successful printing, fruits and vegetables require pre-processing, often involving the addition of hydrocolloids such as starch carriers to achieve the viscoelastic properties necessary for extrusion [7]. The influence of hydrocolloids in fruit-based 3D-printed products has been consistently identified as a key factor affecting product quality, as they play a crucial role in determining printability, structural stability, water distribution, and the retention of bioactive compounds during processing and storage [9].

Although 3D printing has been increasingly applied to fruit-based products, the potential of jostaberry as a functional ingredient in 3D food printing remains largely unexplored. While several berry-based formulations have been investigated, previous studies have mainly focused on printability and formulation characteristics, with limited attention given to the influence of starch type on product quality and the stability of bioactive compounds during storage. Furthermore, information on the physicochemical, antioxidant, and microbiological stability of berry-based 3D-printed products under refrigerated storage remains scarce. Therefore, this study aimed to address these knowledge gaps by evaluating jostaberry-based 3D-printed snacks formulated with corn and wheat starch and assessing their physicochemical properties, bioactive compound stability, antioxidant capacity, and microbiological quality during refrigerated storage. The findings provide new insights into the development of stable, functional 3D-printed fruit products and expand the application potential of this underutilized berry.

Therefore, the primary objective of this study was to assess the suitability of jostaberry as a functional raw material for producing 3D-printed snack products. Particular emphasis was placed on determining how the type of starch carrier (corn vs. wheat) and refrigerated storage time affect physicochemical properties, retention of bioactive compounds and antioxidant capacity, and microbiological stability of the final products. This approach aims to provide formulation and storage insights relevant to developing stable and nutritionally valuable fruit-based 3D-printed foods with antioxidant potential.

Corn and wheat starch were selected in this study because they are among the most commonly used food-grade starches in 3D food printing and have previously been shown to differently influence the quality and stability of printed products. Their distinct physicochemical characteristics may affect moisture retention, texture development, structural integrity, and the stability of bioactive compounds during storage [10]. Previous studies on fruit-based 3D-printed products have demonstrated that starch type can significantly influence physicochemical properties and the retention of phenolic compounds, highlighting the importance of starch selection in the formulation of functional 3D-printed foods [11,12]. Since the objective of this study was to compare their functional performance in the printing matrix rather than their intrinsic structural characteristics, no additional physicochemical characterization of the starches was performed. Therefore, comparing corn and wheat starch provides further insight into their suitability as matrix-forming agents for jostaberry-based 3D printed snacks.

## 2. Materials and Methods

### 2.1. Jostaberry Fruit

Jostaberry fruit (*Ribes × nidigrolaria*) was collected in the northern region of Croatia in June 2024 (Krapina-Zagorje County, Croatia). The fruits were washed, dried and stored in polyvinyl chloride bags at −18 °C. Corn starch (Gustin, Dr. Oetker, Janossomorja, Hungary) and wheat starch (Denes Natura Kft., Pécs, Hungary) were used as structuring agents in the preparation of 3D-printed formulations. These starches were selected due to their widespread industrial use and their distinct physicochemical characteristics, including differences in granule size distribution and amylose–amylopectin ratio, as reported in the literature, which may influence their functional behavior in fruit-based systems.

### 2.2. 3D Printing (3DP) Procedure

#### 2.2.1. Preparation of Fruit Blends for 3D Printing

Prior to analysis, defrosted fruit material was chopped and homogenized using a hand blender (SilverCrest, Bochum, Germany) to prepare the fruit blend. Fruit blends for 3DP were prepared by adding corn or wheat starch (10%, *w*/*w*) to the homogenized jostaberry puree. Several starch concentrations were evaluated during preliminary formulation trials. Concentrations lower than 10% (*w*/*w*) resulted in poor extrusion behavior and inadequate shape retention, whereas concentrations higher than 10% (*w*/*w*) resulted in overly dense mixtures and reduced printability. Therefore, a concentration of 10% (*w*/*w*) was selected for all subsequent experiments.

The prepared blends were heated to 60 °C with continuous mixing to promote partial starch gelatinization and achieve the rheological properties required for successful printing. This temperature was chosen as a moderate processing condition, sufficient to induce starch swelling and viscosity increase while limiting unnecessary thermal degradation of thermolabile bioactive compounds present in jostaberry [13].

#### 2.2.2. 3DP of Jostaberry Fruits

**Figure 1:**
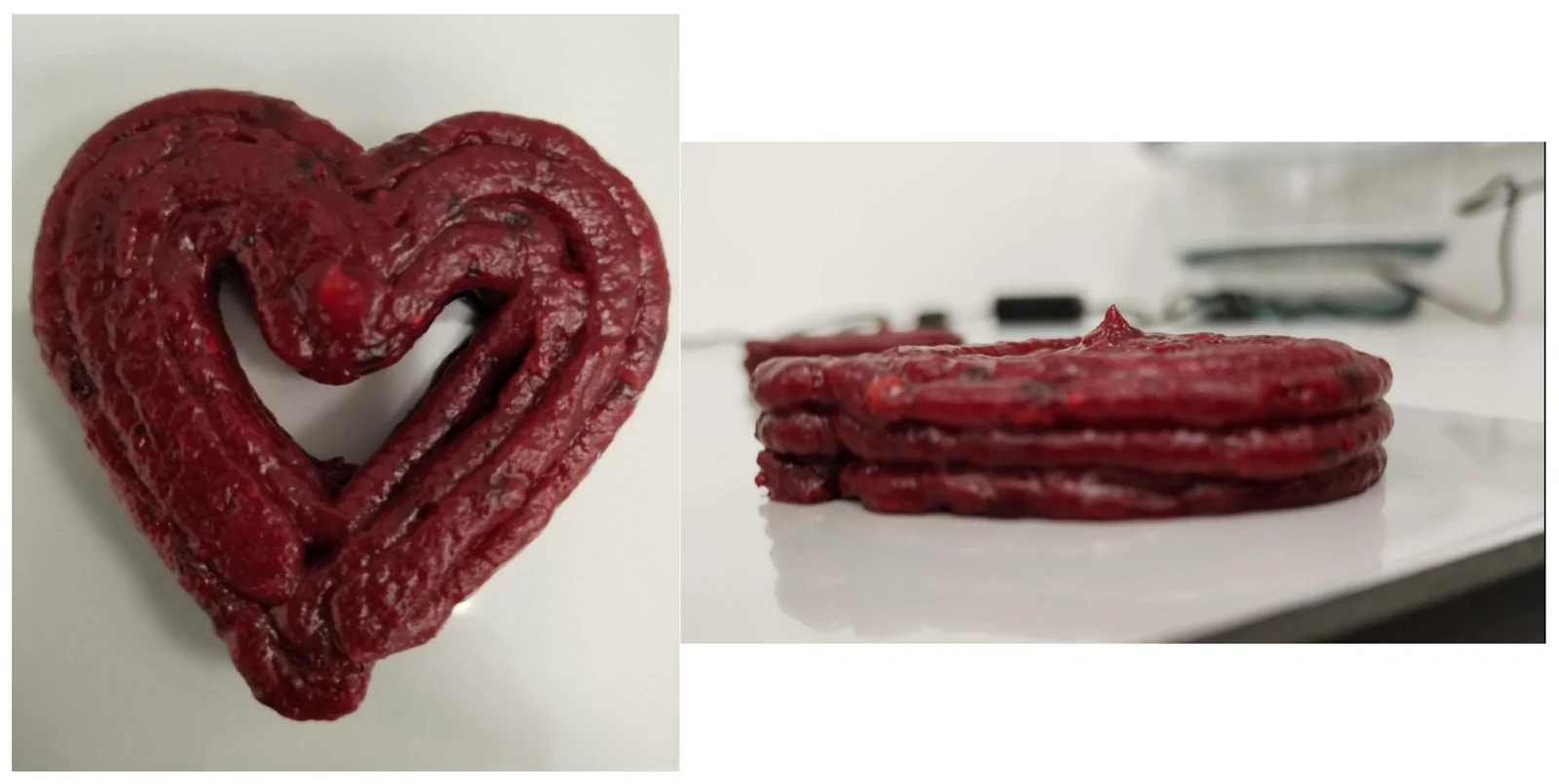
3D-printed jostaberry product with added corn starch (sample J-C-0).

Following the defined experimental design (Table 1), prepared fruit-based blends were extruded by using a commercial extrusion-based system for the 3DP (Foodini^®^ 3D printer, Natural Machines, Barcelona, Spain). Heart-shaped, three-layer 3D-printed products with dimensions of 53 mm (length) × 51 mm (width) × 12 mm (height) (Figure 1) were produced. Printing was performed using the following parameters: nozzle diameter 4 mm, printing speed 1400 mm min^−1^, line thickness 3.4 mm, flow rate 1.65, and first-layer nozzle height 4.5 mm. Freshly prepared 3D-printed products were transferred into sterile Petri dishes and stored at 4 °C in the dark throughout the storage period. Petri dishes remained closed and were opened only during sampling at predefined intervals (0, 2, 5, 8, and 12 days). All samples were handled under identical laboratory conditions during storage and sampling. At each sampling point (0, 2, 5, 8, and 12 days), 3D-printed products were removed from refrigerated storage (4 °C) and prepared immediately for analysis. Prior to all analyses, the printed samples were cut into smaller pieces and homogenized to obtain representative material. All samples were stored under identical refrigerated conditions throughout the experiment to ensure comparability between formulations. Although additional environmental parameters such as relative humidity and oxygen concentration were not independently monitored, these conditions were consistent for all samples and therefore are unlikely to have introduced systematic bias between treatments.

The selected storage duration of 12 days was chosen to represent a realistic refrigerated shelf-life period for fresh 3D-printed fruit-based products intended for commercial application. This timeframe was considered sufficient to monitor progressive changes in physicochemical properties, bioactive compound stability, and microbiological quality under practical storage conditions, while reflecting the expected period during which such products would be stored and consumed. The storage period was also selected to capture both the early and later stages of quality deterioration, thereby providing a comprehensive evaluation of product stability during refrigerated storage.

### 2.3. Physico-Chemical Characterization of the 3D-Printed Products

#### 2.3.1. Determination of Total Dry Matter (TDM), Total Soluble Solids (TSS), and pH

Total dry matter (TDM) content was determined according to the AOAC standard method [14]. Total soluble solids (TSS) were measured using a refractometar (Atago, Pocket, PAL-3, Tokyo, Japan), and results were expressed in Brix (%). TSS were determined to monitor changes in the concentration of soluble constituents during refrigerated storage and to provide an additional indicator of storage-related physicochemical changes in the 3D-printed products. pH values were measured using a pH meter (Mettler-Toledo, Greifensee, Switzerland) in samples diluted with distilled water (1:1, g mL^−1^). All measurements were performed in duplicate.

#### 2.3.2. Determination of CIELab Color Parameters

CIELab color parameters were analyzed using a CIELab colorimeter (Konica Minolta CM-5, Tokyo, Japan), in reflectance mode, with diffuse illumination, 10° viewing angle, illuminant D65, and a target mask of Ø8 mm. Lightness (L*, 0–100), chromatic coordinates a* (−a* = green, +a* = red) and b* chromaticity (−b* = blue, +b* = yellow) were measured, and results were presented as the mean of two replicates. Total color difference (ΔE*), chroma (C*) and hue angle (h°) were calculated according to the following Equations (1)–(3):

(1) $${∁}^{*}=\sqrt{{\left(a^{*}\right)}^{2}+{\left(b^{*}\right)}^{2}}$$

(2) $$h\;[{}^{\circ}]={tan}^{-1}\left(\frac{b^{*}}{a^{*}}\right)$$

(3) $${\Delta E}^{*}=\sqrt{{\left(L^{*}-L_{0}^{*}\right)}^{2}+{\left(a^{*}-a_{0}^{*}\right)}^{2}+{\left(b-b_{0}^{*}\right)}^{2}}$$

L* represents the lightness value of the measured sample, while *L*_0_* refers to the lightness value of the reference or control sample. The difference between L* and *L*_0_* therefore reflects the variation in lightness between the sample under investigation and the reference. Similarly, a* and *a*_0_* describe the difference along the red–green axis, and b* and *b*_0_* the difference along the yellow–blue axis. Taken together, these values define the overall color difference (ΔE*) between the tested and reference samples in the CIELab color space.

#### 2.3.3. HPLC Analysis of Organic Acids

Prior to chromatographic analysis, all samples were filtered through LLG-RC syringe filters with a pore size of 0.45 μm (LLG GmbH, Meckenheim, Germany). The quantification of organic acids was performed according to the procedure described in the literature [15], using an Agilent 1260 Infinity II HPLC system (Agilent Technologies, Waldbronn, Germany), equipped with an autosampler, a thermostatted column compartment, and a diode array detector (DAD). Chromatographic separation was achieved on a COSMOSIL C18-PAQ column (250 × 4.6 mm i.d., 5 μm) maintained at 40 °C. Detection was performed at 245 nm for ascorbic acid and 210 nm for the remaining acids. The injection volume was 20 μL, with a 50 mM phosphate buffer as the mobile phase under isocratic elution conditions at a flow rate of 0.7 mL min^−1^. UV detection was performed at 210 nm for oxalic, malic acid, and citric acid, and at 245 nm for ascorbic acid. Quantification was based on calibration curves prepared with standards of oxalic (10–300 mg L^−1^), malic (10–600 mg L^−1^), ascorbic (0.5–200 mg L^−1^) and citric acid (16–1000 mg L^−1^). The samples were analyzed in triplicate. Results were expressed as mg malic acid (MA), tartaric acid (TA), or ascorbic acid (AA) per 100 g of 3D-printed sample (mg 100 g^−1^). The representative chromatogram has been included in the Supplementary Materials as Figure S1.

### 2.4. Characterization of Bioactive Compounds and Antioxidant Capacity in 3D-Printed Products

#### 2.4.1. Extraction Procedure

Extracts were prepared following a modified protocol for fruit-based 3D-printed samples described in a previous study [12]. Ten grams of the fruit blend or 3D-printed products were mixed with 40 mL of extraction solvent (1% formic acid in 80% methanol, *v*/*v*) in a 100 mL glass beaker, and the mixture was homogenized. The samples were then subjected to ultrasound-assisted extraction using an ultrasonic processor (UP400St, Hielscher Ultrasound Technology, Teltow, Germany) under the following conditions: probe diameter 22 mm, amplitude 50%, pulse 50%, and extraction time 600 s. After extraction, the samples were filtered into a 50 mL volumetric flask, which was subsequently filled to the mark with extraction solvent. The solution was thoroughly mixed and transferred into 50 mL plastic Falcon tubes. The prepared samples were stored at −18 °C until further analyses.

#### 2.4.2. Spectrophotometric Screening of Bioactive Compounds and In Vitro Antioxidant Capacity (AOC)

Spectrophotometric analyses included determination of total phenolic content (TPC), total flavonoids (TF), hydroxycinnamic acid (HCA), condensed tannins (CT), anthocyanins (ANT), as well as antioxidant capacity determined by FRAP and DPPH assays according to protocols for fruit-based 3D-printed samples described in a previous study [12]. Spectrophotometric measurements were performed using an LLG-uniSpec 2 spectrophotometer (LLG GmbH, Meckenheim, Germany). All samples were analyzed in duplicate. The results for TPC, TF, HCA, CT and ANT were expressed as mg of gallic acid equivalents per 100 g of sample (mg GAE 100 g^−1^), mg of quercetin equivalents per 100 g of sample (mg QE 100 g^−1^), mg of chlorogenic acid equivalents per 100 g of sample (mg CAE/100 g), mg of catechin equivalents per 100 g of sample (mg CA 100 g^−1^), and mg of cyanidin-3-galactoside equivalents per 100 g of sample (mg Cyd-3-gal 100 g^−1^), respectively. The results for DPPH (2,2-diphenyl-1-picrylhydrazyl) radical scavenging assay and FRAP (Ferric Reducing Antioxidant Power) test were expressed as mmol of Trolox equivalents per 100 g of sample (mmol TE 100 g^−1^). All measurements were performed in duplicate.

#### 2.4.3. Characterization of Bioactive Compound Profile by HPLC-DAD Analysis

High-performance liquid chromatography (HPLC) was used to determine individual phenolic compounds, following a modified method [16]. Phenolic compounds were analyzed using an Agilent 1260 Infinity II HPLC system (Agilent Technologies, Waldbronn, Germany) equipped with an autosampler, quaternary pump, column thermostat, and diode array detector (DAD). Chromatographic separation was achieved on an Agilent Poroshell 120 SB-C18 column (150 mm × 4.6 mm, 4 μm; Agilent, Palo Alto, CA, USA). The column temperature was maintained at 40 °C for the first 10 min of analysis and then increased to 50 °C from 11 min onward. The mobile phase flow rate was set at 0.8 mL min^−1^, with an injection volume of 20 μL. The mobile phases consisted of solvent A (2% formic acid) and solvent B (methanol), applied under the following gradient program: 0 min, 90:10 (A:B); 10 min, 80:20; 20 min, 70:30; 30 min, 60:40; 40 min, 50:50; 52 min, 10:90; 54 min, 90:10; and 60 min, 90:10. Quantification was based on calibration curves prepared from external standards. Flavonoids were detected at 280, 330, and 360 nm; anthocyanins at 525; hydroxybenzoic acids at 280 nm; and hydroxycinnamic acids at 320 nm. Samples were analyzed in triplicate. The representative chromatograms have been included in the Supplementary Materials as Figures S2 and S3.

### 2.5. Microbiological Analysis

Microbiological analysis was performed on the 3D-printed samples (N = 10) at each predefined storage interval (0, 2, 5, 8 and 12 days) during refrigerated storage at 4 °C. Standardized microbiological methods were applied, and the target microorganisms were selected according to relevant food safety regulations [17]. Detection of *Salmonella* spp. was performed according to ISO 6579-1:2017 [18] using pre-enrichment (peptone-buffered water (PPW), incubation at 37 °C for 16–18 h), selective enrichment (Muller-Kauffmann Tetrathionate-Novobiocin broth (MKTTn), incubation at 37 °C for 24 h, and Rappaport Vassiliadis soya (RVS) peptone broth, incubation at 41.5 °C for 24 h), and plating on selective agar media (Xylose Lysine Deoxycholate (XLD) and Rambach agar, incubation at 37 °C for 24 h). Detection of *Listeria monocytogenes* was performed according to ISO 11290-1:2017 [19] using selective enrichment (Fraser broth, incubation at 37 °C for 48 h), plating on selective agar media (Agar Listeria according to Ottaviani and Agosti (ALOA) medium and Oxford agar, incubation at 37 °C for 48 h).

One gram of each 3D-printed sample was homogenized in 9 mL of PPW broth, and serial dilutions were prepared prior to plating. *Escherichia coli* enumeration was performed according to ISO 7251:2005 [20] using TBX Agar (Tryptone Bile X-glucuronide) incubated at 44 °C for 24 h, followed by colony counting. Coagulase-positive staphylococci (*Staphylococcus aureus* and related species) were enumerated following ISO 6888-1:2021 [21] by surface plating on Baird–Parker agar with confirmation of typical colonies on rabbit plasma (Bio Rad, Hercules, CA, USA). *Clostridium perfringens* counts were determined according to ISO 15213-2:2023 [22] using selective anaerobic agar (Tryptose Sulfite Cycloserine (TSC)) and enumeration methods. *Enterobacteriaceae* were analyzed following ISO 21528-2:2017 [23] using Violet Red Bile Glucose (VRBD) agar, incubated at 37 °C for 24 h, with colony count determination. Yeasts and molds were enumerated according to ISO 21527-2:2008 [24] using selective media, Dichloran Rose-Bengal Chloramphenicol (DRBC), and incubated at 25 °C for 5 days to allow fungal growth. Results were expressed as colony-forming units per gram of sample (CFU g^−1^). All culture media were supplied by Biokar Diagnostic (Allonne, France).

### 2.6. Statistical Analysis

Results of the physicochemical analysis, as well as bioactive characterization and antioxidant capacity, were evaluated by multivariate analysis of variance (MANOVA). The independent variables were (starch type and storage time), while the dependent variables included all measured parameters: physicochemical parameters, bioactive compounds, and antioxidant capacity. Because effect-size measures were not calculated, the present interpretation is based primarily on statistical significance rather than on the magnitude of the observed effects. Post hoc comparisons were performed using Tukey’s test (*p* ≤ 0.05) with Statistica software (ver.12, TIBCO Statistica Ltd., Palo Alto, CA, USA).

## 3. Results and Discussion

### 3.1. Characterization of Physicochemical Properties of 3D-Printed Products

The physicochemical properties and organic acid composition of the 3D-printed products were significantly affected by both starch type and storage duration, as shown in Table 2. The observed changes are discussed in the context of mechanisms previously described for starch-based food systems. However, as the present study did not include rheological or microstructural characterization, the proposed explanations should be considered as literature-supported interpretations rather than direct experimental evidence.

No significant interaction between starch type and storage time was observed (*p* > 0.05), indicating that both factors independently influenced the measured parameters.

The choice of starch (corn vs. wheat) significantly influenced the total soluble solids (TSS) with wheat-based samples showing higher °Bx values (27.13 vs. 25.22 °Bx). This result is consistent with the known differences in granule architecture and amylose-to-amylopectin ratios between corn and wheat starch. Wheat starch typically contains a higher proportion of smaller granules and displays faster swelling and solubilization, which can increase soluble fraction release during processing and storage [25,26].

Although pH was unaffected by starch type (*p* = 0.40), differences were observed in dry matter content, with corn-starch samples exhibiting higher total dry matter (TDM). Corn starch has a higher gelatinization temperature and tends to retain structural rigidity longer during thermal processing, which may reduce water binding and facilitate moisture loss [27].

The concentration of malic acid was significantly influenced by starch type, with wheat formulations showing higher values. The observed differences in malic acid concentrations between starch systems can be related to specific interactions between organic acids and the starch–fruit matrix. Malic acid may exhibit a higher tendency to associate with or be released from the hydrated starch network depending on the microstructural characteristics of the gel. In contrast, tartaric and ascorbic acids did not show significant differences between starch types, indicating that their distribution and stability are less dependent on starch composition and more strongly governed by storage-related chemical processes. This indicates that the behavior of individual organic acids in complex food matrices is compound-specific and cannot be attributed to a single structural feature of starch [28,29,30].

Storage duration exerted a strong influence (*p* ≤ 0.01) on nearly all variables, reflecting the progressive physicochemical and structural evolution of the 3D-printed matrix. TSS exhibited dynamic, non-linear changes, with an initial increase (days 0–2), followed by a decrease (day 5), stabilization (day 8), and a subsequent marked increase by day 12. Such oscillatory behavior is characteristic of starch-based systems undergoing simultaneous moisture migration, partial solubilization, and retrogradation. The early increase in TSS can be attributed to ongoing starch solubilization and the release of low-molecular-weight saccharides, whereas the later increase (day 12) is primarily associated with progressive moisture loss and the consequent concentration of soluble solids [31]. This interpretation is supported by the continuous increase in TDM, from 28.49 ± 0.25% at day 0 to 30.25 ± 0.25% at day 12, indicating gradual dehydration, a well-documented phenomenon in 3D-printed food matrices due to their high surface area-to-volume ratio and porous structure [32]. Similar non-linear changes in TSS solids and moisture-related parameters have been reported in other fruit-based 3D-printed systems, particularly strawberry matrices, where storage-induced water redistribution and matrix restructuring were identified as key drivers of compositional variability [12]. The trends observed in the present study therefore align with established behavior of high-moisture, starch-based printed foods.

From a structural perspective, these changes may be interpreted as indirect indicators of matrix transformations. Moisture loss not only concentrates solutes but also promotes starch retrogradation and matrix densification, leading to redistribution of water and solubilized components. These processes could affect the mobility and release of low-molecular-weight compounds, thereby contributing to the observed variability in TSS [33]. The slight increase and subsequent stabilization of pH could be ascribed to dynamic interactions between organic acids and the evolving starch network, including diffusion limitations, weak buffering effects, and reversible binding or release from the matrix [34]. Collectively, these physicochemical trends reflect underlying microstructural changes, such as reduced porosity, increased polymer–polymer interactions, and altered water distribution. However, it should be emphasized that these relationships are inferred from established principles of starch-based systems, as no direct microstructural or rheological characterization was performed in the present study.

Although the observed trends are consistent with structural changes commonly described in starch-based systems during storage, including starch retrogradation, direct evidence of these processes, such as rheological, textural, or microstructural characterization, was beyond the scope of the present study. Therefore, the proposed mechanisms are based on indirect physicochemical indicators (TSS, moisture loss, and dry weight changes) and are supported by literature describing the evolution of starch gels during storage. Future work should include detailed rheological and structural analyses to confirm the extent and nature of retrogradation in 3D-printed matrices.

All three organic acids exhibited storage-dependent changes, but the patterns differed due to differences in chemical reactivity and stability. Malic acid concentrations significantly decreased from 855.25 ± 12.48 mg 100 g^−1^ at day 0 to 685.50 ± 12.48 mg 100 g^−1^ at day 12. This decline is consistent with previous reports in the literature and may be explained by the combined effects of diffusion-driven redistribution, ionic interactions with starch polymers, and the potential contribution of non-enzymatic browning under acidic conditions [35,36]. Tartaric acid levels fluctuated significantly, with the highest concentration at day 2 (392.50 ± 10.41 mg 100 g^−1^), followed by a steady decrease, reaching the lowest level at day 8 (272.50 ± 10.41 mg 100 g^−1^). In contrast to malic acid, tartaric acid is chemically more stable yet more prone to crystallization and precipitation during moisture migration, a behavior commonly observed as tartrate formation in aqueous/food matrices, which could explain the observed non-linear trend [37].

Ascorbic acid levels showed significant variation, peaking at day 2 (10.25 ± 0.79 mg 100 g^−1^), followed by a progressive decline to the lowest concentration at day 12 (6.75 ± 0.79 mg 100 g^−1^). Degradation of ascorbic acid in acidic matrices is governed by oxidative and hydrolytic pathways, both of which are accelerated by decreasing water activity and the presence of metal ions [38]. The significant effect of storage time (*p* = 0.03) is consistent with progressive oxidative losses typical of 3D-printed or structured food products with large exposed surfaces.

Although comparable structural evolution has been described in other berry-based 3D-printed products, the magnitude of changes observed in this study may be influenced by the specific composition of jostaberry, particularly its high organic acid content and low pH, which can affect starch behavior and water mobility within the matrix.

The visual evaluation of fruit-based 3D-printed products for color stability is an important step in assessing their overall acceptability. The results presented in Table 3 indicate that the type of starch (corn or wheat) did not have a significant effect on the measured color parameters (L*, a*, b*, C*, h*, and ΔE*), as no statistically significant differences were observed (*p* > 0.05). Although corn and wheat starch differ in granule size distribution and amylose–amylopectin ratio, these structural differences were not sufficient to induce measurable changes in color parameters. These results indicate that, under the applied processing conditions, color was predominantly governed by the fruit matrix and pigment composition rather than starch-specific structural features [39,40].

In contrast, storage time significantly affected L* (lightness) and ΔE* (total color difference) (*p* ≤ 0.01), with both parameters showing marked increases after day 5 of storage. The initial decline in L* values (days 0–2) followed by a sharp increase (days 5–12) is consistent with a non-linear structural change in the printed matrix. After prolonged storage, one possible explanation for the increase in L* is starch retrogradation accompanied by microstructural densification, which may enhance light scattering and consequently result in visually lighter surfaces [9]. Although a*, b*, C*, and h* did not reach statistical significance (*p* > 0.05), all showed a progressive upward trend, especially from day 5 onwards. The most pronounced and scientifically relevant trend was observed for ΔE*, which almost tripled between day 0 (1.41) and day 12 (4.27). A ΔE* > 3 is generally considered perceptible to the human eye [41], indicating that storage-driven visual changes were clear and consumer-relevant. Comparable increases in total color difference (ΔE*) during storage have been reported in 3D-printed fruit products, where pigment instability, moisture loss, and matrix densification collectively contribute to visible color changes. Studies on strawberry-based printed products similarly identified storage time as the dominant factor affecting visual quality [12]. The sharp rise in ΔE* after day 5 indicates accelerated physicochemical changes during storage. These mechanisms have been thoroughly described as predominant drivers of surface color modification in starch-rich and printed matrices [42].

Since color is one of the most important sensory attributes influencing consumer perception and acceptance [43], the observed increase in ΔE* during storage could potentially affect the marketability of 3D-printed starch-based products. This is particularly relevant for the development of innovative 3D-printed foods, where preserving visual quality is crucial for consumer acceptance. However, as the measured ΔE* values remained below 6, it can be concluded that the products maintained acceptable color during storage, and the observed changes were not expected to be perceptible to consumers [44].

### 3.2. Bioactive Profile Characterization of 3D Printed Snacks

Bioactive characterization provides important insight into the functional quality of 3D printed snacks, which is strongly influenced by both formulation components and storage conditions. As shown in Table 4, the total phenolic content in jostaberry 3D printed snacks was 225.07 ± 7.88 mg 100 g^−1^. Among the analyzed phenolic subgroups, condensed tannins were the most abundant (181.51 ± 6.24 mg CE 100 g^−1^), followed by anthocyanins (43.46 ± 1.70 mg Cy-3-gal 100 g^−1^) and hydroxycinnamic acids (42.34 ± 1.43 mg CAE 100 g^−1^).

Both starch type and storage duration significantly influenced the stability of bioactive compounds in the products. Formulations containing corn starch exhibited higher levels of total flavonoids and hydroxycinnamic acids compared to wheat-based snacks. These differences can be related to variations in starch structure, including the amylose–amylopectin ratio and microstructural organization, which influence polyphenol–matrix interactions [45]. However, as no direct structural characterization was performed, this interpretation remains inferential. In contrast, starch type did not significantly affect antioxidant capacity. These findings are consistent with previous reports where apparent discrepancy is attributed to the complex nature of starch-based matrices, where viscosity, turbidity, and potential binding of bioactive antioxidants can influence extractability and analytical response, thereby masking compositional differences [46]. Similar trends have been reported in other berry-based 3D-printed products, where starch type influenced the stability of individual phenolic subgroups without significantly affecting total phenolics [11,12]. These effects are generally attributed to non-covalent interactions between starch polymers and phenolic compounds, which depend on starch composition, phenolic structure, and matrix characteristics [47,48]. It should also be noted that wheat and corn starch differ in gelatinization behavior; therefore, processing at a fixed temperature (60 °C) may have resulted in different degrees of gelatinization. Consequently, part of the observed differences between formulations may reflect their distinct thermal responses under identical processing conditions.

The stability of bioactive compounds during storage is a key determinant of the functional quality of 3D-printed jostaberry products. In this study, storage duration exerted a complex influence on phenolic retention and antioxidant capacity. While total phenolic content remained stable over 12 days, all individual phenolic subgroups showed a decreasing trend. Total flavonoids declined by 25.8% (from 12.03 to 8.93 mg QE 100 g^−1^), whereas hydroxycinnamic acids decreased by 8.0% (from 42.28 to 38.78 mg CAE 100 g^−1^).

The degradation of flavonoids (8.1%) and hydroxycinnamic acids (27.5%) can be attributed to oxidative mechanisms. Hydroxycinnamic acids are particularly susceptible to radical-mediated reactions, including structural cleavage, decarboxylation, and de-esterification, leading to the formation of lower-molecular-weight phenolics or polymerized derivatives with reduced extractability [49,50]. Similarly, flavonoids undergo enzymatic and non-enzymatic oxidation, resulting in quinone formation and subsequent polymerization or degradation [51]. Comparable trends have been reported in other berry-based 3D-printed systems, particularly strawberry matrices, where phenolic losses during storage were linked to oxidative and structural factors [12].

Among the analyzed compounds, condensed tannins and anthocyanins exhibited the greatest decline, decreasing by 27.6% and 37.3%, respectively. The pronounced instability of anthocyanins is primarily associated with their sensitivity to oxygen, pH, and temperature, which promote structural degradation and polymerization [52]. The reduction in condensed tannins is likewise consistent with oxidative polymerization and condensation reactions leading to the formation of high-molecular-weight complexes with limited extractability [53]. Although such mechanisms are consistent with observations in starch-based systems, where matrix restructuring affects compound accessibility and mobility [42], it should be noted that these interpretations are based on indirect evidence, as no direct microstructural characterization was performed in this study.

It should also be considered that starch–polyphenol interactions are bidirectional. In addition to the influence of starch on the retention and extractability of phenolic compounds, polyphenols may also modify starch functionality by interacting with amylose and amylopectin through hydrogen bonding and hydrophobic interactions. These interactions have been reported to affect starch gelatinization, retrogradation, crystallinity, and water mobility, thereby influencing the physicochemical stability of starch-based systems during storage. Although such mechanisms could also contribute to the changes observed in the present study, they were not directly investigated, as no rheological, textural, or microstructural analyses were performed [54].

In parallel with the compositional changes occurring during storage, significant alterations in antioxidant capacity were also observed. FRAP values decreased by 27.9%, closely mirroring the reduction in phenolic content and confirming the central role of phenolics in antioxidant capacity. In contrast, DPPH values remained relatively stable, consistent with the retention of radical-scavenging activity by certain phenolic subclasses or partial compensation by non-phenolic antioxidants for the observed losses. Overall, storage beyond eight days resulted in a decreased stability of bioactive antioxidants. These findings are in agreement with previous reports describing time-dependent oxidation and polymerization of phenolics in starch-based systems [55] and highlight the importance of matrix composition and storage conditions in preserving bioactive stability in 3D-printed fruit products.

From a practical perspective, the observed reductions in antioxidant compounds became substantial mainly after 8 days of refrigerated storage. Although the products remained microbiologically safe and visually acceptable throughout the entire 12-day storage period, the marked decline in anthocyanins, condensed tannins, and FRAP values after day 8 indicates a progressive loss of antioxidant functionality. Therefore, while the products can be considered technologically stable for up to 12 days under refrigerated conditions, storage beyond 8 days may compromise their nutritional value as a functional food. Similarly, although ΔE* increased significantly during storage, the values remained below the threshold generally considered unacceptable for consumers. Therefore, the observed color changes are unlikely to limit consumer acceptance under the investigated storage conditions.

To provide deeper insight into storage-induced changes, the stability of individual phenolic compounds was comprehensively characterized, enabling identification of compound-specific degradation patterns (Table 5). Among the identified compounds, anthocyanins were predominant, with cyanidin-3-*O*-rutinoside (13.93 mg 100 g^−1^), cyanidin-3-*O*-glucoside (6.45 mg 100 g^−1^), and delphinidin derivatives present in comparatively high concentrations. In contrast, hydroxybenzoic and hydroxycinnamic acids (2.12 mg 100 g^−1^) and kaempferol (0.53 mg 100 g^−1^) were detected at substantially lower levels. Despite increasing interest in functional foods, the polyphenolic composition of jostaberry remains insufficiently characterized, with existing studies reporting variable levels of anthocyanins, phenolic acids, flavanols, flavonols, and tannins [3,5,6].

Starch type did not significantly affect most hydroxybenzoic and hydroxycinnamic acids (*p* > 0.05), reflecting the limited sensitivity of these low-molecular-weight compounds to the botanical origin of starch. This observation is consistent with previous findings indicating weak interactions between simple phenolic acids and amylose–amylopectin structures, resulting in relatively uniform distribution across starch matrices [56]. An exception was observed for ferulic acid, which was significantly higher in corn-based samples (*p* = 0.02), consistent with its greater native abundance in corn cell wall polysaccharides compared to wheat [57].

In contrast, anthocyanins exhibited a clear dependence on starch type. Wheat-based snacks contained significantly higher levels of cyanidin glycosides (*p* ≤ 0.01), which can be attributed to differences in starch structural properties. Wheat starch typically forms more cohesive gel networks, which may enhance the retention and stability of polar, cationic anthocyanins during processing and storage [58,59]. Conversely, kaempferol content remained unaffected by starch type, supporting the notion that flavonol–starch interactions are weaker and highly structure-dependent [60].

Storage duration emerged as the dominant factor affecting phenolic composition. A progressive decline was observed for several compounds, including kaempferol (−66%), *p*-coumaric acid (−40%), and chlorogenic acid (−60%). The decrease in chlorogenic acid was particularly pronounced (*p* ≤ 0.01), indicating ongoing degradation or transformation during storage. These trends are consistent with known oxidative and radical-mediated degradation pathways of phenolic compounds, involving cleavage, isomerization, and polymerization reactions [61,62].

In contrast to the overall declining trend, anthocyanins remained comparatively stable throughout storage, with the exception of cyanidin-3-*O*-glucoside, which displayed a non-linear behavior characterized by an increase in concentration until day 8, followed by stabilization or a slight decline. Structural rearrangements within the starch matrix may weaken starch–polyphenol interactions and facilitate the release of previously bound anthocyanins. Similar effects have been reported in starch-based systems, where matrix restructuring alters water mobility and compound accessibility [63]. However, this interpretation remains indirect, as no structural characterization was performed.

The acidic environment (pH 2.7–2.8) can further contribute to these changes. Acid-catalyzed hydrolysis of starch can induce partial depolymerization, weakening the matrix and increasing molecular mobility. Therefore, the observed trends are consistent with previous reports and support the interpretation that these changes result from the combined effects of retrogradation and acid-induced hydrolysis rather than from a single mechanism [64]. Following day 8, anthocyanin levels declined, which is consistent with the establishment of equilibrium between bound and free fractions, followed by gradual degradation. The relatively high stability of these compounds compared to kaempferol can be attributed to glycosylation, which enhances resistance to degradation. In contrast, flavonols are more susceptible to oxidative breakdown under acidic conditions [52,65].

In conclusion, the findings align with previously reported behavior of berry-based 3D-printed products, particularly regarding storage-induced physicochemical changes and phenolic instability. However, the distinct compositional features of jostaberry, including its high acidity and specific polyphenolic profile, appear to modulate these processes, highlighting the importance of matrix-specific interactions in determining phenolic stability in 3D-printed snacks.

### 3.3. Microbiological Quality of 3DP Products

Microbiological analysis of the 3D-printed snacks was performed at each predefined storage interval (0, 2, 5, 8, and 12 days) during refrigerated storage at 4 °C (Table 6). No pathogenic bacteria, yeasts, or molds were detected in any of the analyzed samples, indicating satisfactory microbiological safety under the applied storage conditions, which is in line with the prescribed regulations for foodstuffs [66]). An exception was noted in sample J-C-12 (corn starch), where two visible mold colonies were observed on the surface prior to analysis. These colonies had not undergone sporulation and were aseptically removed; consequently, their identification was not possible. Their occurrence is attributable to incidental post-processing contamination, presumably originating from airborne fungal propagules during storage. These findings are consistent with previous research on 3D-printed fruit-based systems, such as the study by Bebek Markovinović et al. [12], which reported the absence of pathogenic microorganisms in 3D-printed strawberry snacks throughout refrigerated storage, supporting the microbiological safety of such products.

Despite the absence of detectable microorganisms at the end of storage, the lack of intermediate time-point analyses limits insight into microbial growth dynamics. Therefore, the results should be interpreted as evidence of short-term microbiological stability rather than a definitive assessment of shelf life.

The observed stability can be primarily attributed to intrinsic product characteristics. The low pH of the samples (~2.7) creates an unfavorable environment for the growth of most spoilage and pathogenic microorganisms, while the presence of phenolic compounds contributes additional antimicrobial effects. These intrinsic factors, in combination with refrigerated storage, collectively govern microbial inhibition.

## 4. Conclusions

This study demonstrated that both starch type and refrigerated storage significantly influenced the physicochemical properties, bioactive profile, and antioxidant stability of 3D-printed jostaberry snacks. Differences between formulations prepared with corn and wheat starch confirmed that starch selection plays an important role in determining product characteristics during storage. Although the mechanisms underlying these differences were not directly investigated, the observed trends are consistent with the distinct physicochemical properties of the starches reported in the literature.

From a practical perspective, the findings provide useful guidance for the development of stable fruit-based 3D-printed foods intended for refrigerated storage. Starch selection and storage duration should be carefully considered during product formulation, as both factors affected physicochemical characteristics and the retention of bioactive compounds. Under the applied storage conditions, the products maintained satisfactory microbiological quality and acceptable physicochemical stability for up to 12 days, indicating their potential suitability for short-term commercial distribution.

Several limitations should be acknowledged. The present study compared the functional performance of two commercially available starches without direct structural characterization; therefore, mechanistic explanations of starch-matrix interactions are based on literature and should be interpreted with caution. Furthermore, although samples were stored under controlled refrigerated conditions (4 °C, in the dark, in closed Petri dishes), environmental humidity and oxygen concentration inside the containers were not continuously monitored, which may have contributed to minor variability during storage. In addition, only a single starch concentration was evaluated, limiting the ability to generalize the observed effects.

Despite these limitations, the comparative design of the study, in which both starch formulations were processed and stored under identical experimental conditions, provides a reliable basis for evaluating the relative effects of starch type and storage duration on the physicochemical properties and bioactive stability of 3D-printed jostaberry snacks.

Overall, this study provides new insights into the behavior of starch-based 3D-printed fruit systems during refrigerated storage and contributes to the development of functional 3D-printed foods with improved quality and shelf stability. Future research should include structural characterization of starches, rheological and microstructural analyses of printed matrices, evaluation of different starch concentrations, optimization of storage conditions, and sensory assessment to further support the industrial application of these systems.

Overall, the present findings suggest that refrigerated storage for up to 8 days provides the best balance between technological stability and retention of bioactive compounds, whereas storage up to 12 days remains acceptable from a food safety and technological standpoint.

Future research should incorporate rheological analysis, texture profile analysis, microscopy, and water-mobility measurements to directly assess starch–matrix and starch–polyphenol interactions and to validate the mechanisms proposed in the present study.

## Figures and Tables

**Table 1:** Experimental design for jostaberry-based 3D printed snacks.

Sample	Starch Type	Storage Time (Days)	Sample ID
Jostaberry fruit blend	/	/	J
3D-printed jostaberry products	Corn	0	J-C-0
	Wheat	0	J-W-0
	Corn	2	J-C-2
	Wheat	2	J-W-2
	Corn	5	J-C-5
	Wheat	5	J-W-5
	Corn	8	J-C-8
	Wheat	8	J-W-8
	Corn	12	J-C-12
	Wheat	12	J-W-12

**Table 2:** Influence of starch type and storage duration on the physicochemical properties and organic acid content of 3D printed snacks.

Variable	N	TSS (°Bx)	pH	TDM (%)	Malic Acid	Tartaric Acid	Ascorbic Acid
Formulation		*p* ≤ 0.01 *	*p* = 0.40 **	*p* ≤ 0.01 *	*p* = 0.09 **	*p* = 0.74 **	*p* = 0.28 **
Corn	10	25.22 ± 0.32 ^a^	2.76 ± 0.00 ^a^	29.71 ± 0.16 ^b^	753.00 ± 7.89 ^a^	323.50 ± 6.58 ^a^	9.20 ± 0.50 ^a^
Wheat	10	27.13 ± 0.32 ^b^	2.76 ± 0.00 ^a^	28.77 ± 0.16 ^a^	773.10 ± 7.89 ^b^	326.70 ± 6.58 ^a^	8.40 ± 0.50 ^a^
Storage (days)		*p* ≤ 0.01 *	*p* ≤ 0.01 *	*p* ≤ 0.01 *	*p* ≤ 0.01 *	*p* ≤ 0.01 *	*p* = 0.03 **
0	4	25.30 ± 0.50 ^a^	2.73 ± 0.00 ^a^	28.49 ± 0.25 ^a^	855.25 ± 12.48 ^a^	351.50 ± 10.41 ^a^	9.50 ± 0.79
2	4	26.63 ± 0.50 ^a^	2.76 ± 0.00 ^b^	28.73 ± 0.25 ^ab^	779.00 ± 12.48 ^b^	392.50 ± 10.41 ^ae^	10.25 ± 0.79 ^c^
5	4	24.55 ± 0.50 ^a^	2.77 ± 0.00 ^cb^	29.10 ± 0.25 ^ab^	765.00 ± 12.48 ^cb^	330.50 ± 10.41 ^af^	7.75 ± 0.79 ^b^
8	4	25.45 ± 0.50 ^a^	2.77 ± 0.00 ^db^	29.63 ± 0.25 ^bc^	730.00 ± 12.48 ^dbc^	272.50 ± 10.41 ^b^	9.75 ± 0.79 ^b^
12	4	28.95 ± 0.50 ^b^	2.78 ± 0.00 ^ecd^	30.25 ± 0.25 ^c^	685.50 ± 12.48 ^ed^	278.50 ± 10.41 ^cb^	6.75 ± 0.79 ^a^
Dataset average	20	26.18 ± 0.46	2.76 ± 0.00	29.24 ± 0.20	763.05 ± 13.96	325.10 ± 11.11	8.80 ± 0.44

TSS—Total Soluble Solids; TDM—Total Dry Matter. Organic acids are expressed in mg 100 g^−1^ of sample. Results are expressed as mean ± standard error. Values represented with the different letters are statistically different at *p* ≤ 0.05; * significant factor in multifactor analysis; ** not a significant factor in multifactor analysis.

**Table 3:** Influence of starch type and storage duration on the color parameters of 3D printed snacks.

Variable	n	L*	a*	b*	C*	h*	ΔE*
Starch type		*p* = 0.92 **	*p* = 0.70 **	*p* = 0.45 **	*p* = 0.66 **	*p* = 0.32 **	*p* = 0.19 **
Corn	10	15.06 ± 0.19 ^a^	27.17 ± 0.56 ^a^	7.87 ± 0.29 ^a^	28.30 ± 0.62 ^a^	16.09 ± 0.31 ^a^	2.70 ± 0.19 ^a^
Wheat	10	15.03 ± 0.19 ^a^	27.49 ± 0.56 ^a^	8.20 ± 0.29 ^a^	28.69 ± 0.62 ^a^	16.55 ± 0.31 ^a^	3.06 ± 0.19 ^a^
Storage (days)		*p* ≤ 0.01 *	*p* = 0.09 **	*p* = 0.07 **	*p* = 0.08 **	*p* = 0.13 **	*p* ≤ 0.01 *
0	4	14.08 ± 0.30 ^a^	26.76 ± 0.89 ^a^	7.61 ± 0.46 ^a^	27.83 ± 0.97 ^a^	15.87 ± 0.49 ^a^	1.41 ± 0.30 ^a^
2	4	13.95 ± 0.30 ^a^	26.06 ± 0.89 ^a^	7.40 ± 0.46 ^a^	27.10 ± 0.97 ^a^	15.86 ± 0.49 ^a^	1.63 ± 0.30 ^a^
5	4	15.51 ± 0.30 ^b^	28.46 ± 0.89 ^a^	8.36 ± 0.46 ^a^	29.67 ± 0.97 ^a^	16.37 ± 0.49 ^a^	2.99 ± 0.30 ^b^
8	4	16.09 ± 0.30 ^b^	29.18 ± 0.89 ^a^	9.23 ± 0.46 ^a^	30.61 ± 0.97 ^a^	17.55 ± 0.49 ^a^	4.09 ± 0.30 ^b^
12	4	15.58 ± 0.30 ^b^	26.18 ± 0.89 ^a^	7.57 ± 0.46 ^a^	27.26 ± 0.97 ^a^	15.95 ± 0.49 ^a^	4.27 ± 0.30 ^b^
Dataset average	20	15.04 ± 0.23	27.33 ± 0.44	8.04 ± 0.24	28.49 ± 0.50	16.32 ± 0.24	2.88 ± 0.30

Results are expressed as mean ± standard error. Values represented with the different letters are statistically different at *p* ≤ 0.05; * significant factor in multifactor analysis; ** not a significant factor in multifactor analysis.

**Table 4:** Influence of starch type and storage duration on the bioactive compounds and antioxidant capacity of 3D printed snacks.

Variable	N	TPC	TF	HCA	CT	ANT	FRAP	DPPH
Starch type		*p* = 0.12 **	*p* ≤ 0.01 *	*p* ≤ 0.01 *	*p* = 0.51 **	*p* = 0.06 **	*p* = 0.35 **	*p* = 0.54 **
Corn	10	232.18 ± 6.13 ^a^	12.35 ± 0.37 ^a^	48.00 ± 0.69 ^a^	183.53 ± 4.22 ^a^	44.94 ± 1.01 ^a^	1.78 ± 0.05 ^a^	0.28 ± 0.00 ^a^
Wheat	10	217.96 ± 6.13 ^a^	10.43 ± 0.37 ^b^	36.69 ± 0.69 ^b^	179.50 ± 4.22 ^a^	41.98 ± 1.01 ^a^	1.72 ± 0.05 ^a^	0.28 ± 0.00 ^a^
Storage (days)		*p* ≤ 0.01 *	*p* ≤ 0.01 *	*p* = 0.03 *	*p* ≤ 0.01 *	*p* ≤ 0.01 *	*p* ≤ 0.01 *	*p* ≤ 0.01 *
0	4	243.56 ± 9.70 ^a^	12.03 ± 0.58 ^a^	42.28 ± 1.09 ^a^	184.30 ± 6.67 ^a^	48.13 ± 1.60 ^a^	1.79 ± 0.07 ^a^	0.28 ± 0.00 ^a^
2	4	235.72 ± 9.70 ^a^	11.97 ± 0.58 ^a^	43.21 ± 1.09 ^a^	199.10 ± 6.67 ^a^	47.61 ± 1.60 ^a^	1.85 ± 0.07 ^a^	0.28 ± 0.00 ^a^
5	4	228.57 ± 9.70 ^a^	11.21 ± 0.58 ^ab^	43.28 ± 1.09 ^a^	189.56 ± 6.67 ^a^	44.41 ± 1.60 ^a^	1.99 ± 0.07 ^a^	0.28 ± 0.00 ^a^
8	4	249.56 ± 9.70 ^a^	12.82 ± 0.58 ^a^	44.18 ± 1.09 ^ab^	201.02 ± 6.67 ^a^	46.94 ± 1.60 ^a^	1.85 ± 0.07 ^a^	0.27 ± 0.00 ^b^
12	4	167.96 ± 9.70 ^a^	8.93 ± 0.58 ^b^	38.78 ± 1.09 ^ac^	133.59 ± 6.67 ^b^	30.19 ± 1.60 ^b^	1.29 ± 0.07 ^b^	0.28 ± 0.00 ^a^
Dataset average	20	225.07 ± 7.88	11.39 ± 0.44	42.34 ± 1.43	181.51 ± 6.24	43.46 ± 1.70	1.75 ± 0.06	0.28 ± 0.00

TPC—total phenolic content (mg GAE 100 g^−1^); TF—total flavonoid content (mg QE 100 g^−1^); HCA—total hydroxycinnamic acids (mg CAE 100 g^−1^); CT—total condensed tannins (mg CE 100 g^−1^); ANT—total monomeric anthocyanins (mg Cy-3-gal 100 g^−1^); ABTS assay (mmol TE 100 g^−1^), FRAP assay (mmol TE 100 g^−1^); DPPH assay (mmol TE 100 g^−1^). Results are expressed as mean ± standard error. Values represented with the different letters are statistically different at *p* ≤ 0.05; * significant factor in multifactor analysis; ** not a significant factor in multifactor analysis.

**Table 5:** Influence of starch type and storage duration on individual phenolic compounds of 3D-printed products.

Variable	n	Dihydroxy-benzoic Acid	Hydroxybenzoic Acid	Chlorogenic Acid	Caffeic Acid	Coumaric Acid	Ferulic Acid	Kaempferol	Cyanidin-3-O-glucoside	Cyanidin-3-O-rutinoside	Delphinidin-3-O-glucoside	Delphinidin-3-O-rutinoside
Starch type		*p* = 0.18 **	*p* = 0.46 **	*p* = 0.87 **	*p* = 0.26 **	*p* = 0.26 **	*p* = 0.02 *	*p* = 0.08 **	*p* ≤ 0.01 *	*p* ≤ 0.01 *	*p* = 0.69 **	*p* = 0.51 **
Corn	10	0.46 ± 0.12 ^a^	0.60 ± 0.12 ^a^	0.40 ± 0.01 ^a^	0.34 ± 0.02 ^a^	0.09 ± 0.00 ^a^	0.08 ± 0.00 ^a^	0.57 ± 0.03 ^a^	5.98 ± 0.16 ^a^	12.80 ± 0.47 ^a^	0.85 ± 0.06 ^a^	6.59 ± 0.27 ^a^
Wheat	10	0.69 ± 0.12 ^a^	0.73 ± 0.12 ^a^	0.40 ± 0.01 ^a^	0.31 ± 0.02 ^a^	0.10 ± 0.00 ^a^	0.06 ± 0.00 ^b^	0.48 ± 0.03 ^a^	6.91 ± 0.16 ^b^	15.06 ± 0.47 ^b^	0.82 ± 0.06 ^a^	6.33 ± 0.27 ^a^
Storage (days)		*p* = 0.10 **	*p* = 0.05 *	*p* ≤ 0.01 *	*p* = 0.01 *	*p* ≤ 0.01 *	*p* = 0.01 *	*p* ≤ 0.01 *	*p* ≤ 0.01 *	*p* = 0.07 **	*p* = 0.57 **	*p* = 0.38 **
0	4	0.41 ± 0.19 ^a^	0.89 ± 0.19 ^a^	0.48 ± 0.01 ^a^	0.30 ± 0.03 ^a^	0.10 ± 0.00 ^a^	0.06 ± 0.01 ^a^	0.98 ± 0.05 ^a^	5.06 ± 0.26 ^a^	11.79 ± 0.74 ^a^	0.86 ± 0.09 ^a^	5.84 ± 0.43 ^a^
2	4	0.72 ± 0.19 ^a^	1.07 ± 0.19 ^ab^	0.45 ± 0.01 ^a^	0.40 ± 0.03 ^ab^	0.14 ± 0.00 ^bd^	0.10 ± 0.01 ^b^	0.46 ± 0.05 ^b^	5.65 ± 0.26 ^a^	14.17 ± 0.74 ^a^	0.96 ± 0.09 ^a^	7.06 ± 0.43 ^a^
5	4	1.00 ± 0.19 ^a^	0.67 ± 0.19 ^a^	0.44 ± 0.01 ^a^	0.35 ± 0.03 ^a^	0.10 ± 0.00 ^a^	0.06 ± 0.01 ^ac^	0.43 ± 0.05 ^cb^	7.04 ± 0.26 ^b^	14.85 ± 0.74 ^a^	0.79 ± 0.09 ^a^	6.25 ± 0.43 ^a^
8	4	0.48 ± 0.19 ^a^	0.47 ± 0.19 ^a^	0.34 ± 0.01 ^b^	0.36 ± 0.03 ^ad^	0.08 ± 0.00 ^a^	0.06 ± 0.01 ^a^	0.43 ± 0.05 ^db^	8.44 ± 0.26 ^c^	14.62 ± 0.74 ^a^	0.81 ± 0.09 ^a^	6.64 ± 0.43 ^a^
12	4	0.26 ± 0.19 ^a^	0.22 ± 0.19 ^ac^	0.29 ± 0.01 ^c^	0.21 ± 0.03 ^ac^	0.06 ± 0.00 ^c^	0.08 ± 0.01 ^a^	0.33 ± 0.05 ^eb^	6.05 ± 0.26 ^a^	14.22 ± 0.74 ^a^	0.76 ± 0.09 ^a^	6.53 ± 0.43 ^a^
Dataset average	20	0.58 ± 0.10	0.66 ± 0.10	0.40 ± 0.02	0.32 ± 0.02	0.09 ± 0.01	0.07 ± 0.00	0.53 ± 0.06	6.45 ± 0.31	13.93 ± 0.46	0.84 ± 0.04	6.46 ± 0.19

* Results are expressed as mean ± standard error. Values represented with the different letters are statistically different at *p* ≤ 0.05; * significant factor in multifactor analysis; ** not a significant factor in multifactor analysis.

**Table 6:** Microbiological quality of 3D-printed jostaberry snacks after 12 days of storage at 4 °C.

Sample	*Salmonella* sp. *	*Enterobacteriaceae* **	*Staphylococcus aureus* **	*Clostridium perfringens* **	*Listeria monocytogenes* **	Yeasts and Molds **	*Escherichia coli* **
J	0	<10	<10	<10	0	<50	<10
J-C-0	0	<10	<10	<10	0	<50	<10
J-W-0	0	<10	<10	<10	0	<50	<10
J-C-2	0	<10	<10	<10	0	<50	<10
J-W-2	0	<10	<10	<10	0	<50	<10
J-C-5	0	<10	<10	<10	0	<50	<10
J-W-5	0	<10	<10	<10	0	<50	<10
J-C-8	0	<10	<10	<10	0	<50	<10
J-W-8	0	<10	<10	<10	0	<50	<10
J-C-12	0	<10	<10	<10	0	<50	<10
J-W-12	0	<10	<10	<10	0	<50	<10

* Expressed in CFU/25 g; ** expressed in CFU/g. (a) *Salmonella* sp.—0, (b) *Enterbacteriaceae*—1 × 10^3^, (c) *S. aureus*—1 × 10^2^, (d) *C. perfringens*—1 × 10^4^, (e) *L. monocytogenes*—0, (f) yeasts and molds—1 × 10^3^, (g) *E. coli*. The explanation of the sample codes (J, J-C-0, J-W-0, J-C-2, J-W-2, …, J-C-12, and J-W-12) is provided in Table 1.

## Data Availability

The original contributions presented in this study are included in the article. Further inquiries can be directed to the corresponding author.

## Supplementary Materials

The following supporting information can be downloaded at https://www.mdpi.com/article/10.3390/antiox15080971/s1, Figure S1: HPLC chromatogram of anthocyanins at 525 nm detected in the 3D-printed sample: 1- delphinidin-3-rutinoside, 2- cyanidin-3-rutinoside, 3- delfinidin-3-glucoside, 4- cyanidin-3-glucoside; Figure S2: HPLC chromatogram of hydroxycinnamic acids at 330 nm detected in the 3D-printed sample: 5- chlorogenic acid, 6- caffeic acid, 7- p-coumaric acid, and 8- ferulic acid; Figure S3: HPLC chromatogram of organic acids at 210 nm detected in jostaberry fruit blend: 9-tartaric acid, 10-malic acid, 11-ascorbic acid, 12-citric acid.
