# Supplementary material for: Jostaberry (Ribes × nidigrolaria) as a Functional Ingredient for 3D Printed Snacks: Impact of Starch Type and Storage on Antioxidant Profile and Stability
Source: Antioxidants (Basel). 2026 Aug 5;15(8):971. doi: 10.3390/antiox15080971 (PMC13509390; doi:10.3390/antiox15080971)
Supplement: Supplementary file 1 [file antioxidants-15-00971-s001.zip › antioxidants-4407704-supplementary.pdf]

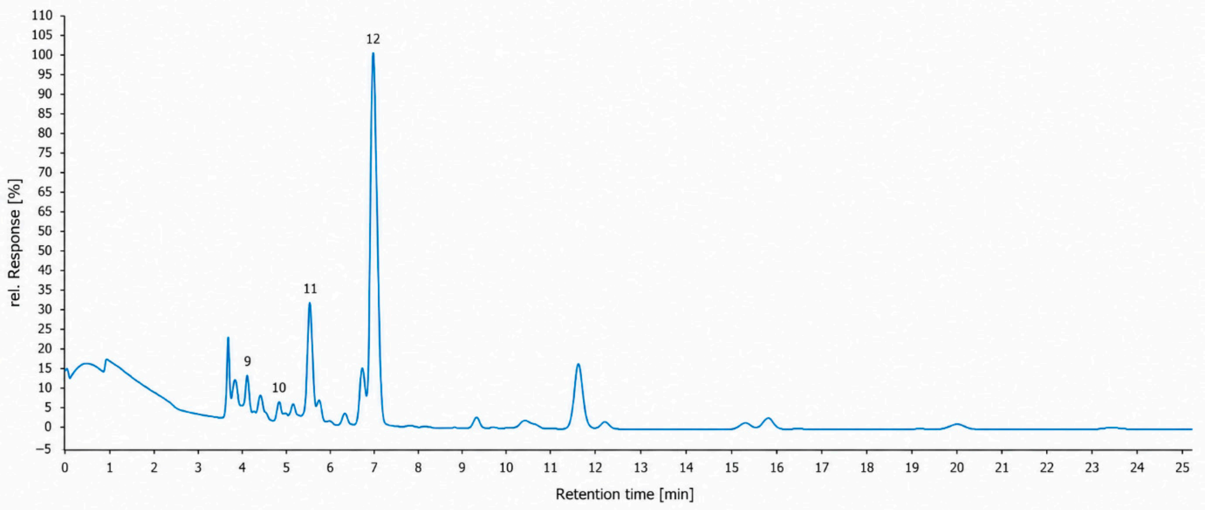

Figure S1. HPLC chromatogram of organic acids at 210 nm detected in jostaberry fruit blend: 9-tartaric acid, 10-malic acid, 11-ascorbic acid, 12-citric acid.

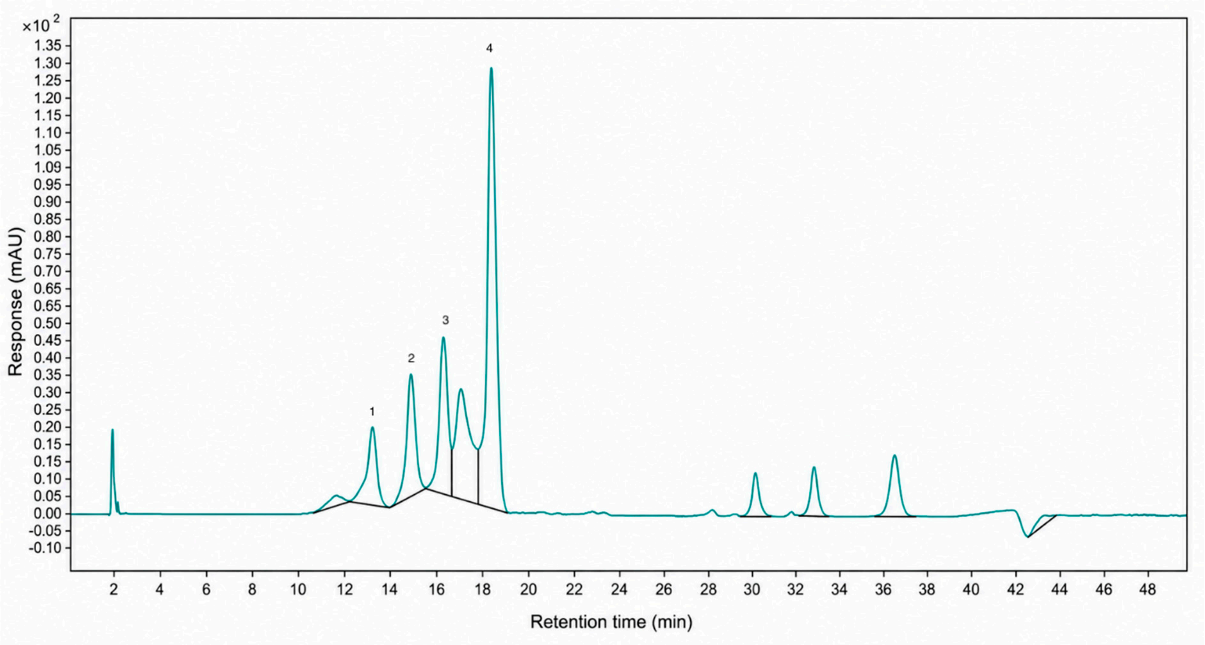

Figure S2. HPLC chromatogram of anthocyanins at 525 nm detected in the 3D-printed sample: 1-delphinidin-3-rutinoside, 2- cyanidin-3-rutinoside, 3- delfinidin-3-glucoside, 4- cyanidin-3-glucoside.

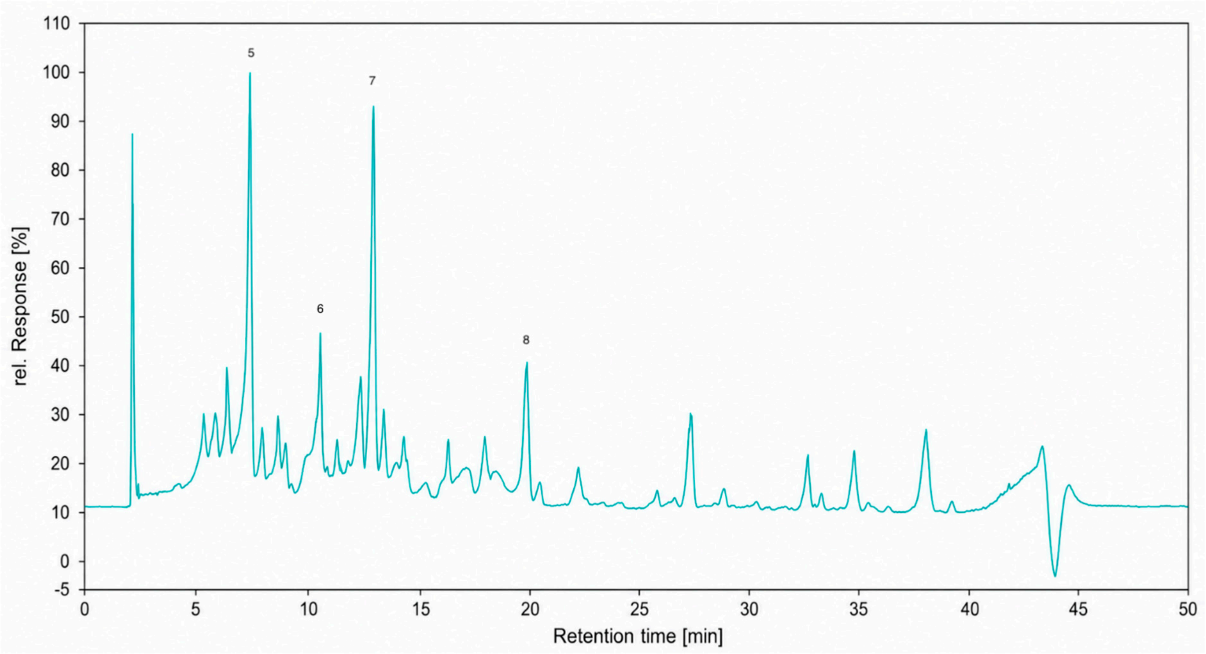

Figure S3. HPLC chromatogram of hydroxycinnamic acids at 330 nm detected in the 3D-printed sample: 5 – chlorogenic acid, 6 – caffeic acid, 7 – *p*-coumaric acid, and 8 – ferulic acid.
